# Authenticated Semi-Quantum Key Distribution Protocol Based on W States

**DOI:** 10.3390/s22134998

**Published:** 2022-07-02

**Authors:** Hung-Wen Wang, Chia-Wei Tsai, Jason Lin, Chun-Wei Yang

**Affiliations:** 1 Master Program for Digital Health Innovation, College of Humanities and Sciences, China Medical University, No. 100, Sec. 1, Jingmao Rd., Beitun Dist., Taichung 406040, Taiwan; u109217001@cmu.edu.tw; 2 Department of Computer Science and Information Engineering, National Taichung University of Science and Technology, No. 129, Sec. 3, Sanmin Rd., North Dist., Taichung 40401, Taiwan; cwtsai@nttu.edu.tw; 3 Department of Computer Science and Engineering, National Chung Hsing University, No. 145, Xingda Rd., South Dist., Taichung 40227, Taiwan; jasonlin@nchu.edu.tw

**Keywords:** authentication, semi-quantum key distribution, w state, quantum cryptography

## Abstract

In 2019, Wen et al. proposed authenticated semi-quantum key distribution (ASQKD) for identity and message using the teleportation of W states and GHZ-like states without pre-shared keys. However, the ASQKD protocol presents a vital issue in the teleportation of W states owing to its inappropriate design. Bob recovers the teleported W states without obtaining the position of the corresponding photons and then returns the recovered photons back to Alice. Hence, the teleportation of W states in Wen et al.’s ASQKD protocol was malfunctioning. Moreover, Wen et al.’s ASQKD protocol requires quantum memory, which strongly disobeys the definition of semi-quantum proposed by Boyer et al. Therefore, in this study, we discover the flaws of Wen et al.’s ASQKD protocol and propose an authenticated semi-quantum key distribution protocol. When compared to Wen et al.’s ASQKD protocol, the proposed ASQKD protocol has the following advantages: legal semi-quantum environment (i.e., does not require quantum memory), reduced quantum hardware requirement (i.e., based only on W states), does not involve classical cryptography (i.e., the hash function), and provided 1.6 times higher qubit efficiency.

## 1. Introduction

To ensure security, applications perform encryption techniques to secure data. Mainstream encryption based on prime factorization mostly relies on public-key cryptography to distribute secured keys. However, in 1994, Shor [1] proposed a quantum algorithm for determining the prime factors of an integer in polynomial time, which revealed the insecurity of popular public-key cryptography (PKC) systems, such as RSA encryption. This revelational breakthrough indicated the unsafe cryptography of classical computers and led to the research on quantum cryptography. Thus, the design of a protocol that can withstand quantum computer attacks has become an important topic in quantum cryptography.

In 1984, Bennett and Brassard proposed the first quantum key distribution (QKD) protocol based on single photons [2]. The QKD protocols distribute keys between two participants based on quantum mechanics. Participants can detect eavesdropping during transmission by using quantum states. Eventually, a secret key is shared using quantum and authenticated classical channels. QKD protocols have been adapted to various versions based on different security and environmental issues. Since then, researchers conducted research on quantum cryptography (i.e., quantum secure direct communication [3,4,5,6,7] and quantum secret sharing [8,9,10,11,12]). However, QKD protocols assume an authenticated classical channel between Alice and Bob (i.e., the transmitted classical messages can be eavesdropped upon but not modified). If an authenticated classical channel does not exist between Alice and Bob, then QKD protocols can suffer from an impersonation attack. Mutual identity authentication is required to prevent impersonation attacks in QKD protocols. Therefore, authenticated QKD (AQKD) protocols [13,14,15] have been proposed to circumvent these security problems.

However, the AQKD protocols [13,14,15] mentioned above typically assume that both participants possess full quantum capabilities. Quantum hardware, which is prohibitively expensive, is still in the development phase. Hence, assuming that all participants have full quantum capabilities is not practical. In light of this, in 2007, Boyer et al. [16] proposed the semi-quantum key distribution (SQKD) protocol. Boyer et al. [17] defined an environment that involves two types of users: suppose one user (Alice) obtains full quantum capabilities, and another user (Bob) retains classical capabilities with limited quantum capabilities. Bob can perform three operations by the following abilities: (1) measuring qubits in Z-basis (i.e., {|0〉, |1〉}); (2) preparing Z-basis qubits; (3) using delay line to reorder qubits; and (4) reflecting qubits without any disturbance. Boyer et al. [17] defined two schemes for SQKD protocols: randomization-based and measure-resend. In the randomization SQKD protocol, Bob can perform (1) Z-basis measurement, (3) reordering qubits through delay lines, and (4) reflecting qubits without any disturbance. In the measure-resend SQKD protocol, Bob can perform (1) Z-basis measurements, (2) prepare Z-basis qubits, and (4) reflect qubits without any disturbance. After the proposal, miscellaneous protocols have been applied within a “semi” environment. For example, SQKD protocols [18,19,20,21,22,23,24,25,26,27,28], semi-quantum secret sharing protocols [29,30,31,32,33,34,35,36,37,38,39], semi-quantum secure direct communication [40,41,42,43,44,45,46,47,48,49], semi-quantum key agreement [50,51,52,53], and semi-quantum private comparison [54,55,56,57,58].

In 2014, Yu et al. [59] presented the first authenticated semi-quantum key distribution (ASQKD) protocol that does not require authenticated classical channels. By pre-sharing a master secret key between two communicants, a sender with advanced quantum devices can transmit a working key to a receiver who can only perform classical operations. The concept of ASQKD enables the establishment of a key hierarchy in security systems that also eases key management problems. In 2016, Li et al. [60] presented two advanced ASQKD protocols. When compared with Yu et al.’s [59] ASQKD protocols, the proposed protocols ensure better qubit efficiency and require fewer pre-shared keys. In 2016, Meslouhi and Hassouni [61] identified a vulnerability that allows a malicious person to recover a partial master key and launch a successful man-in-the-middle attack. In 2020, Tsai and Yang [62] proposed a lightweight authenticated semi-quantum key distribution (LASQKD) protocol. By pre-sharing a master key and adopting a one-way communication strategy, the proposed protocol allows a quantum user and classical user to share secret keys without using an authenticated classical channel or a Trojan horse detection device. In 2020, Zwbboudj et al. [63] presented a new ASQKD protocol without entanglement, which can realize higher security than the schemes of Yu et al. [59] and Li et al. [60]. The proposed scheme is also simpler and demands less advanced quantum devices than ASQKD schemes that use entanglement. In 2021, Chang et al. [64] proposed a new measure-resend ASQKD protocol. The proposed ASQKD protocol uses only single photons, requires fewer pre-shared keys, and provides better qubit efficiency than state-of-the-art ASQKD protocols. However, an eavesdropper can launch a reflective attack to forge the receiver’s identity without being detected. In addition, Chang et al.’s ASQKD protocol assumes an authenticated classical channel between the sender and the receiver. It is considered illogical to have an authenticated channel in the ASQKD protocol. Therefore, in 2022, Wang et al. [65] proposed an efficient and secure ASQKD protocol to circumvent these problems using only single photons.

In 2019, Wen et al. [66] proposed a authenticated semi-quantum key distribution for message and identity based on W state and GHZ-like state. Wen et al.’s ASQKD protocol exhibits the following advantages when compared to other related protocols:

(1) It reduces the quantum hardware equipment when compared to other ASQKD protocols.
(2) It does not require pre-share keys.
(3) Wen et al. demonstrated that the proposed ASQKD protocol is robust against typical attacks.
(4) It is highly efficient than some of the existing ASQKD protocols.

Although Wen et al.’s ASQKD protocol is highly efficient and secure, in this study, we discover the design flaws of Wen et al.’s ASQKD protocol as follows: (1.) Wen et al.’s ASQKD protocol is impossible to execute. In Wen et al.’s ASQKD protocol, Bob recovers the teleportation of W states without obtaining the positions of the corresponding qubits. Theoretically, Alice and Bob cannot perform a security check on the transmission qubits because Bob is unable to perform teleportation of the W state appropriately. (2.) Wen et al.’s ASQKD protocol requires quantum memory, which strongly disobeys the semi-quantum definition of Boyer et al. [17]. Hence, in this study, we propose an ASQKD protocol based only on the W states. When compared to Wen et al.’s ASQKD protocol [66], the proposed ASQKD protocol has several advantages.

1. The proposed ASQKD protocol ensures the procedure is functional.
2. The proposed ASQKD protocol does not require quantum memory and legally fulfills a semi-quantum environment [17].
3. The proposed ASQKD protocol, based on W states, only reduces the quantum hardware requirements.
4. The qubit efficiency of the proposed ASQKD protocol is 1.6 times higher than that of Wen et al.’s ASQKD protocol.
5. The proposed ASQKD protocol does not require classical cryptography (i.e., the hash function), which does not show the potential menace of the advance quantum computing.

The remainder of this paper is organized as follows. Section 2 provides a review of Wen et al.’s ASQKD protocol. Section 3 describes the security issues associated with Wen et al.’s ASQKD protocol. Section 4 describes the proposed measure-resend ASQKD protocol. Section 5 presents security analysis. Section 6 presents efficiency analysis. Finally, conclusions of the study are stated in Section 7.

## 2. Review of Wen et al.’s ASQKD Protocol

Wen et al.’s ASQKD protocol, based on W states and GHZ-like states, allows quantum user Alice and classical user Bob to authenticate messages and identities mutually within the semi-quantum environment. In Wen et al.’s ASQKD protocol, Alice possesses all quantum capabilities, whereas Bob is treated as a classical user who can only perform measurements on a Z-basis, preparing Z-basis qubits, and reflecting qubits without disturbing. To initiate the protocol, Alice and Bob must pre-share a secret specific bases set,$\;\{|\kappa^{+}\rangle ,|\kappa^{-}\rangle ,|\gamma^{+}\rangle ,|\gamma^{-}\rangle \}$. Alice and Bob mark these four bases. When Alice measures the qubits, she announces the notation ($\left\{\kappa^{+},\kappa^{-},\gamma^{+},\gamma^{-}\right\}$) as opposed to the quantum basis to Bob. Alice and Bob determine the notation. They notify each other of the changes according to one binary string. The binary string includes nine numbers. The first number denotes the order of change, sequential or reverse. The remaining numbers indicate the notation and corresponding bases. The corresponding notation changes after each successful authentication process. Eventually, Alice will send the state string |Y〉, and the authentication information to Bob. |Y〉 will be conveyed by teleportation of W state. Wen et al.’s ASQKD protocol is as follows:

Step W1. Alice prepares *n* GHZ-like states as shown in Equation (1) and divides these states into three sequences: $S_{a},S_{b},S_{c}$. Every photon in $S_{a}$ represents all the first particles in GHZ-like states, and $S_{b}$ and $S_{c}$ represent all the second and third particles in GHZ-like states. Then, she inserts random decoy states {|0〉,|1〉,|+〉,|−〉} into $S_{a}$ and obtains ${S^{\prime }}_{a}$ as follows:
  (1) $$\begin{array}{ll} |G\rangle_{abc} & =\frac{1}{2}{\left(|001\rangle +|010\rangle +|100\rangle +|111\rangle \right)}_{abc}\\ & =\frac{1}{2}\left({|0\rangle }_{a}{|\psi^{+}\rangle }_{bc}+{|1\rangle }_{a}{|\phi^{+}\rangle }_{bc}\right)\; \end{array}$$

Alice prepares 2n W states, as in Equation (2), and divides these states into three sequences: $S_{1}$,$\;S_{2}$,$\;S_{3}$. Photons in $S_{1}$ include all the first particles in the W state. Similarly,$\;S_{2}$ and $S_{3}$ include all the second and third particles in the W state.

(2) $$|W\rangle_{123}=\frac{1}{2}\left(|100\rangle +|010\rangle +\sqrt{2}|001\rangle \right)$$

Suppose one of the four W bases is selected as follows:

(3) $$|\kappa^{\pm }\rangle =\frac{1}{2}\left(|010\rangle +|001\rangle \pm \sqrt{2}|100\rangle \right)$$

(4) $$|\gamma^{\pm }\rangle =\frac{1}{2}\left(|110\rangle +|101\rangle \pm \sqrt{2}|000\rangle \right)$$

These bases are utilized to measure W states’ first and second particles ($\left|W\rangle_{12}\right)$ and a single particle *m*,$\;|\varphi \rangle_{m}={\left(\alpha |0\rangle +\beta |1\rangle \right)}_{m}$. Then, the measurement result is as follows:

$$\begin{array}{l} \left|\varphi \rangle_{m}\right|W\rangle_{123}\\ =(\alpha \left|0\rangle +\beta \right|1\rangle {)}_{m}⊗\frac{1}{2}(\left|100\rangle +\right|010\rangle +\sqrt{2}|001\rangle {)}_{123}\\ =\frac{1}{2}[\alpha (\left|010\rangle +\right|001\rangle {)}_{m12}\left|0\rangle_{3}+\sqrt{2}\alpha \right|000\rangle_{m12}|1\rangle_{3}\\ +\beta (\left|110\rangle +\right|101\rangle {)}_{m12}|0\rangle_{3}+\sqrt{2}\beta |100\rangle_{m12}|1\rangle_{3}]\\ =\frac{1}{2}[|\kappa^{+}\rangle_{m12}(\alpha |0\rangle +\beta |1\rangle {)}_{3}+|\kappa^{-}\rangle_{m12}(\alpha \left|0\rangle -\beta \right|1\rangle {)}_{3}\\ +|\gamma^{+}\rangle_{m12}(\alpha \left|1\rangle +\beta \right|0\rangle {)}_{3}+|\gamma^{-}\rangle_{m12}(-\alpha \left|0\rangle +\beta \right|1\rangle {)}_{3}) \end{array}$$

Eventually, Alice sends ${S^{\prime }}_{a}$ and $S_{3}$ to Bob.

Step W2. Alice encodes state string |Y〉 according to the specific coding rule (see also Table 1), generates a binary message string $L=\{L_{i}|i=1,2,\ldots ,2n.\}$, then recodes the binary string L according to the following rules: binary message {0,1} recodes into the Z-basis {|0〉,|1〉}. Eventually, Alice obtains the new particle string |$\varphi_{L}\rangle$ = {$|\varphi_{L_{j}}\rangle \;|\;j=1,\;2,\;\ldots ,2n.$}. Alice performs W-basis measurement on $\left(|Y\rangle {,\;|S}_{1}\rangle {,\;|S}_{2}\rangle \right)$ and $\left(|\varphi_{L}\rangle ,{\;|S}_{1}\rangle {,\;|S}_{2}\rangle \right)$ and performs Bell measurement on $(\left|S_{b}\rangle ,\right|S_{c}\rangle ).$ Then, Alice can obtain the measurement results $MR_{Y},MR_{L},MR_{Bell},$ respectively. Alice informs Bob of the measurement results via a classical channel. It should be noted that $MR_{Y},MR_{L}$ is presented in notation format $\{|\kappa^{+}\rangle ,|\kappa^{-}\rangle ,|\gamma^{+}\rangle ,|\gamma^{-}\rangle \}.$

Step W3. Bob receives the measurement results, $MR_{Y},\;MR_{L}$ from Alice. He can perform the unitary operation on $S_{3}$ to recover the states $|Y\rangle \;\mathrm{and}\;|\varphi_{L}\rangle$ based on the measurement result $MR_{Y},\;MR_{L}$. Bob measures $|\varphi_{L}\rangle$ in $S_{3}$ using a Z-basis. Then, he records the measurement results as $M_{3}=\left\{m_{3}^{1},m_{3}^{2},\ldots \ldots ,m_{3}^{2n}\right\}$. According to the notation and the measurement result of $M_{3}$, Bob applies the corresponding coding rule and obtains $M_{3}^{\prime }=\left\{{m^{\prime }}_{3}^{1},\;{m^{\prime }}_{3}^{2},\ldots ,\;{m^{\prime }}_{3}^{2n}\right\}$ as shown in Table 2 below.

Step W4. Bob obtains |Y〉 and $M_{3}^{\prime }$ and returns $|Y^{\prime }\rangle$ based on $M_{3}^{\prime }.$ If $M_{3}^{\prime }=00$ or $M_{3}^{\prime }=01$, then he measures |Y〉 in the Z-basis, prepares the same photon as $|Y^{\prime }\rangle$, and resends it back to Alice. If $M_{3}^{\prime }=10$ or $M_{3}^{\prime }=11$, then Bob returns |Y〉 as $|Y^{\prime }\rangle$ directly to Alice. Furthermore, Alice checks the received decoy states using the correct corresponding basis.

Step W5. Alice measures $|Y^{\prime }\rangle$ on the correct basis and checks if $|Y^{\prime }\rangle$ equals to the original |Y〉. Alice then announces the position of $|\varphi_{L}\rangle$ and decoy photons in $S_{a}^{\prime }$ to Bob via the classical channel. According to this announcement, Bob removes the decoy state in $S_{a}^{\prime }$ and recovers sequence $S_{a}.$ Then, Bob measures $S_{a}$ on the Z-basis to check its correlation with $MR_{Bell}$.

Step W6. Based on the measurement results of $S_{a}$, Bob generates binary string $L_{B}=\{L_{B_{j}}\;|j=1,\;2,\;\ldots ,\;2n\}$ according to the following coding rules: if the measurement result is |0〉, then he encodes $L_{B_{j}}=00$. Furthermore, while the measurement result is |1〉, he encodes $L_{B_{j}}=01$. Bob hashes $L_{B}$ to obtain the hash value *H*($L_{B}$). Bob then sends *H*($L_{B}$) to Alice.

Step W7. Alice calculates *H*(L) and checks if *H*($L_{B}$) equals *H*(L).

## 3. Security Issues in Wen et al.’s ASQKD Protocol

Wen et al.’s ASQKD protocol proved security analysis under popular attacks. However, the protocol suffers from vital flaws in the procedure. Hence, it can be considered as a malfunctioning protocol. Moreover, the protocol requires classical Bob to equip quantum memory, which strongly disobeys the principle of the semi-quantum environment as stated by Boyer et al. [17]. The issues in the teleportation of W states and quantum environment are described as follows.

### 3.1. Teleportation of W States in Wen et al.’s ASQKD Protocol

In Wen et al.’s ASQKD protocol, teleportation of W states between Alice and Bob is a vital procedure flaw. In Step W1, Bob receives ${S^{\prime }}_{a}$ and $S_{3}$ from Alice. In Step W3, Bob recovers corresponding photons in $S_{3}$ into $|Y\rangle \;\mathrm{and}\;|\varphi_{L}\rangle$ based on $MR_{Y},MR_{L},$ and the measurement result of $S_{3}$. In Step W4, Bob returns $|Y^{\prime }\rangle$ to Alice. It should be noted that Alice announces the position of $|\varphi_{L}\rangle$ in Step W5, and it is inferred that Bob does not obtain any position of $|Y\rangle \;\mathrm{and}\;|\varphi_{L}\rangle$ in line with $S_{3}$ in Step W3. This implies that the insufficient information on the corresponding position of $|Y\rangle \;\mathrm{and}\;|\varphi_{L}\rangle$ cannot allow Bob to distinguish between $|Y\rangle \;\mathrm{and}\;|\varphi_{L}\rangle$. Thus, Bob cannot perform any recovery in Step W3, which eventually leads to the failure of the teleportation of W states. Hence, Wen et al.’s ASQKD protocol teleportation of W states cannot be performed under all circumstances.

### 3.2. Quantum Environment Issue in Wen et al.’s ASQKD Protocol

In Wen et al.’s ASQKD protocol, Alice sends photons ($S_{a}^{\prime }$ and $S_{3}$) to Bob in Step W1, and Bob obtains all received photons. Then, Bob receives the measurement results from Alice in Step W2. In Step W4, Bob performs corresponding unitary operations on each photon in $S_{3}$, recovers $S_{3}$ into $|Y\rangle \;\mathrm{and}\;|\varphi_{L}\rangle$ based on $MR_{Y},MR_{L},$ and the measurement result of $S_{3}$, respectively. Eventually, Bob returns $|Y^{\prime }\rangle$ to Alice. The interval between receiving the photons (Step W1), measuring the $S_{3}$ photon sequence, calculating and performing the recovery based on notations and measurement results (Step W3), and returning $|Y^{\prime }\rangle$ based on $M_{3}^{\prime }$ (Step W4) obviously requires quantum memory to store the photons for performing all the procedures until resending back to Alice. Hence, the classical Bob in Wen et al.’s ASQKD protocol is equipped with quantum memory, which strongly disobeys the definition of a semi-quantum environment [17].

## 4. Proposed Measure-Resend ASQKD Protocol

**Figure 1:**
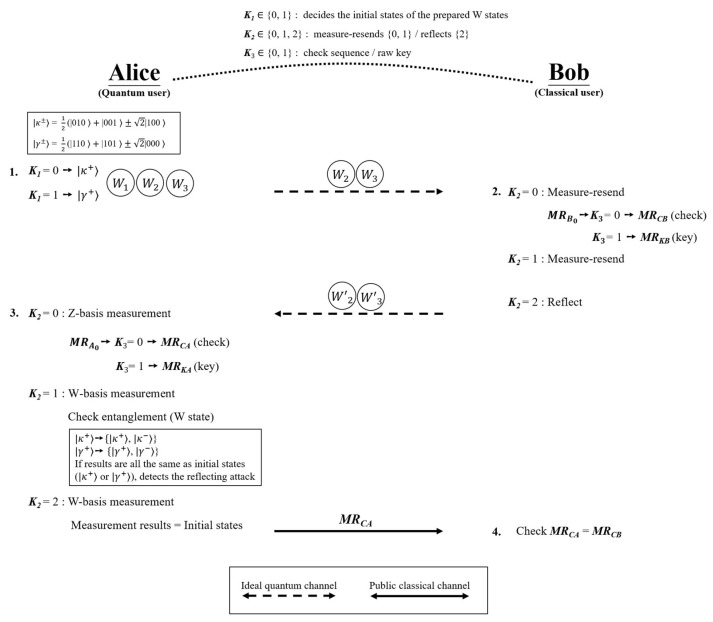
Proposed ASQKD protocol.

The proposed ASQKD protocol ensures that the quantum environment fulfills the definition of semi-quantum, which was defined by Boyer et al. [17] by using W states only. Assume that Alice is a quantum user and Bob is a classical user with limited quantum capabilities. Alice and Bob pre-share three binary keys, $K_{1},K_{2}K_{3}$**.** Specifically, $K_{1}$ determines the initial state of the prepared W state and $K_{2}$ represents measure-resending or reflecting photons. $K_{3}$ determines the photon to be the check sequence or key sequence. Figure 1 illustrates the proposed scheme. The steps involved in the proposed ASQKD protocol are as follows:

Step 1. Alice prepares the initial W states based on $K_{1}.$ If $K_{1}=0$, then Alice prepares $|\kappa^{+}\rangle$, and while $K_{1}=1$, she prepares $|\gamma^{+}\rangle$. Alice then divides the W states into three sequences: $W_{1}$, $W_{2}$***,*** and $W_{3}.$ $W_{1}$ represents all the first particles of W states. Similarly, $W_{2}$ and $W_{3}$ represent all the second and third particles of W states, respectively. Alice sends $W_{2}$ and $W_{3}$ to Bob one photon at a time.

Step 2. For every received photon, Bob performs measure-resending or reflects photons based on $K_{2}$.
  - If $K_{2}=0$, then Bob measures the received photon on a Z-basis, prepares the same photon as the measurement result, and resends it to Alice. For the measured sequence at $K_{2}=0$, if $K_{3}=0$, then Bob records the measurement results to the check sequence $MR_{CB}$; if $K_{3}=1$, then Bob records the measurement results to the key sequence $MR_{KB}$.
  - If $K_{2}=1$, then Bob measures the received photon on a Z-basis, prepares the same photon as the measurement result, and resends it to Alice.
  - If $K_{2}=2$, then Bob reflects the received photon back to Alice without any influence.

Step 3. Alice receives $W_{2}^{\prime }$ and $W_{3}^{\prime }$ from Bob. She performs Z-basis or W-basis measurements based on $K_{2}$.
  - If $K_{2}=0$, then Alice performs a Z-basis measurement and classifies it into two measured sequences based on $K_{3}$. If $K_{3}=0$, then Alice records the measurement results as a check sequence $MR_{CA}$, whereas if $K_{3}=1$, Alice records the measurement results to the key sequence $MR_{KA}$.
  - If $K_{2}=1$, then Alice performs a W-basis measurement to check the entanglement correlation of the W states. Hence, according to the uncertainty principle, if the initial state is $|\kappa^{+}\rangle$ or $|\gamma^{+}\rangle$, then the measured states should collapse into $\{|\kappa^{+}\rangle ,\;|\kappa^{-}\rangle$} or $\{|\gamma^{+}\rangle ,\;|\gamma^{-}\rangle$}. Otherwise, if the states remain the same as the initial state, then it is inferred that Bob does not measure the photons and Alice will detect that the protocol may suffer from the reflecting attack.
  - If $K_{2}=2$, then Alice performs a W-basis measurement for the eavesdropping check. Alice compares the measurement results with the initial states. This implies that if the states remain the same as the original states, neither Bob nor Eve measure the photons, proving the security of the transmission.
  After the eavesdropping check, Alice announces $MR_{CA}$ to Bob.

Step 4. Bob checks if $MR_{CA}=MR_{CB}$ to secure the channel. Eventually, Alice and Bob share a raw key as the measurement result of $MR_{KA},MR_{KB}$ (i.e., if one measures |00〉,|01〉,|10〉,|11〉, represents classical bits “00”, “01”, “10”, “11”, respectively.). Then, they perform a privacy amplification process [67,68] on the raw key to distill the private key.

## 5. Security Analysis

In this section, the security of the proposed ASQKD protocol is analyzed with respect to the five main attacks.

### 5.1. Impersonation Attack

#### 5.1.1. Assume Eve Essayed to Impersonate Alice

Suppose Eve attempts to impersonate Alice. In Step 1, Eve may generate photon sequences, $E_{2}\;\mathrm{and}\;E_{3}$, to impersonate the photons sent by Alice, $W_{2}\;\mathrm{and}\;W_{3}.$ Bob receives $E_{2}\;\mathrm{and}\;E_{3}$, performs the measure-resend mode or the reflected mode based on $K_{2}$, obtains $MR_{CB}\;\mathrm{and}\;MR_{KB}$. In Step 4, Alice announces the check sequence $MR_{CA}$. After Bob receives it, he verifies Alice by collating the check sequence with *MR_CB_*. Bob detects eavesdropping because $MR_{CB}\neq MR_{CA}.$ Eve cannot impersonate any check sequence *MR_CA_* because it was generated based on $K_{3}$, which was previously pre-shared privately. Hence, Eve cannot successfully impersonate Alice in the proposed ASQKD protocol.

#### 5.1.2. Assume Eve Essayed to Impersonate Bob

Suppose that Eve attempts to impersonate Bob. Eve may generate photons $E_{2}^{\prime }\;\mathrm{and}\;E_{3}^{\prime }$ to impersonate $W_{2}^{\prime }\;\mathrm{and}\;W_{3}^{\prime }$. In Step 3, Alice performs an eavesdropping check. She measured $E_{2}^{\prime }\;\mathrm{and}\;E_{3}^{\prime }$ to check the entanglement of the W states. According to the uncertainty principle, if $K_{2}=1$, then the measure-resend photons should collapse into $\{|\kappa^{+}\rangle ,\;|\kappa^{-}\rangle$} or $\{|\gamma^{+}\rangle ,\;|\gamma^{-}\rangle$}; if $K_{2}=2$, then the reflected state should be the same as the initial state. Thus, if the states are not related to the correct procedure based on $K_{2}$, then Alice detects impersonation of Eve.

### 5.2. Reflecting Attack

If Eve tries to perform the reflecting attack, then Eve may intercept the photon sequences $W_{2}\;\mathrm{and}\;W_{3}$ in Step 1 and subsequently resend the same photons back to Alice (i.e., $W_{2}\;\mathrm{and}\;W_{3}$). For every received photon in $W_{2}\;\mathrm{and}\;W_{3}$, Alice performs an eavesdropping check in Step 3 based on $K_{2}$. If Bob measure-resends $W_{2}\;\mathrm{and}\;W_{3}$, then the state should collapse into $\{|\kappa^{+}\rangle ,\;|\kappa^{-}\rangle$} or $\{|\gamma^{+}\rangle ,\;|\gamma^{-}\rangle$} according to the uncertainty principle. As mentioned, the photon sequence $W_{2}\;\mathrm{and}\;W_{3}$ is reflected by Eve and not measured by Bob, which does not collapse into $\{|\kappa^{+}\rangle$, $|\kappa^{-}\rangle$} or $\{|\gamma^{+}\rangle$,$|\gamma^{-}\rangle$}. From the perspective of Alice, if the state remains in the same state as the initial state, then she can infer that Bob does not measure any photons and Alice detects that the protocol is suffering from the reflecting attack.

### 5.3. Man-in-the-Middle Attack

Assume that Eve attempts to perform a man-in-the-middle attack on the transmitted photon between Alice and Bob. In Step 1, Eve may intercept $W_{2}\;\mathrm{and}\;W_{3}$, in which the initial states of W states are $\{|\kappa^{+}\rangle ,\;|\gamma^{+}\rangle$}. Then, Eve measures $W_{2}\;\mathrm{and}\;W_{3}$ to obtain the information, and eventually, Eve generates photon sequences $E_{2}\;\mathrm{and}\;E_{3}$ based on the measurement results and sends them to Bob. It should be noted that the photon sequence $E_{2}\;\mathrm{and}\;E_{3}$ collapses into the random state $\{|\kappa^{+}\rangle ,\;|\kappa^{-}\rangle$} or $\{|\gamma^{+}\rangle ,\;|\gamma^{-}\rangle$}. In Step 2, Bob measure-resends or reflects $E_{2}\;\mathrm{and}\;E_{3}$ based on $K_{2}$, namely $E_{2}^{\prime }\;\mathrm{and}\;E_{3}^{\prime }$. After receiving $E_{2}^{\prime }\;\mathrm{and}\;E_{3}^{\prime },$ Alice proceeds with an eavesdropping check. If Eve can pass the eavesdropping check in Step 3, then she is able to successfully perform a man-in-the-middle attack. However, without knowing the pre-shared key $K_{2}$, Eve cannot compute the check sequence for the reflected photons. Once Eve measures $W_{2}\;\mathrm{and}\;W_{3}$, it can successfully pass the eavesdropping check with a probability of 1/2. Hence, the probability of Eve being detected in the proposed ASQKD protocol is $1-{\left(\frac{1}{2}\right)}^{\frac{n}{3}}$. While *n* is large enough, the detection probability is approximately 100%. Thus, in Step 3, Alice detects that the ASQKD protocol is attacked by Eve in Step 3.

### 5.4. Collective Attack

This section proves the proposed ASQKD protocol is immune to collective attack and does not show leakage if there is no error detected.

In the attack scheme, let Eve obtains all quantum abilities and gains the control of quantum channels. Eve may try to eavesdrop on private information from Alice and Bob through the collective attack. To initiate the attack, Eve prepares a probe qubit $|E_{1}\rangle$ and operates a unitary operation on the joint state |q〉, the qubits which transmit through the quantum channel. Eve performs the $U_{1}$ operation in Step 1, Eve entangles the probe qubits with the traveling qubit which sent by Alice. Then, Eve performs the $U_{2}$ operation in Step 2 and entangles the probe qubits with the transmitting qubit which is resent by Bob.

**Theorem** **1.** *Eve can operate the collective attack to eavesdrop on private information without being detected. To initiate the attack, Eve performs the first unitary operation* $U_{1}$ *operation on the qubits sent by Alice. Then, Eve performs the second unitary operation* $U_{2}$ *operation on the qubits which are resent by Bob. However, no operation can provide Eve to deduce the private information without being detected.*

**Proof** **of** **Theorem 1.** Assume Eve operates a unitary operator to eavesdrop on the qubit sent from Alice in Step 1 by $U_{1}$, the possibilities are presented as follows:

$$U_{1}\left(|00\rangle ⊗|E_{1}\rangle \right)=a_{0}|00\rangle |g_{A0}\rangle +a_{1}|01\rangle |g_{A1}\rangle +a_{2}|10\rangle |g_{A2}\rangle +a_{3}|11\rangle |g_{A3}\rangle \;U_{1}\left(|01\rangle ⊗|E_{1}\rangle \right)=b_{0}|00\rangle |h_{A0}\rangle +b_{1}|01\rangle |h_{A1}\rangle +b_{2}|10\rangle |h_{A2}\rangle +b_{3}|11\rangle |h_{A3}\rangle \;U_{1}\left(|10\rangle ⊗|E_{1}\rangle \right)=c_{0}|00\rangle |i_{A0}\rangle +c_{1}|01\rangle |i_{A1}\rangle +c_{2}|10\rangle |i_{A2}\rangle +c_{3}|11\rangle |i_{A3}\rangle \;U_{2}\left(|00\rangle ⊗|E_{1}\rangle \right)=d_{0}|00\rangle |j_{A0}\rangle +d_{1}|01\rangle |j_{A1}\rangle +d_{2}|10\rangle |j_{A2}\rangle +d_{3}|11\rangle |j_{A3}\rangle \;U_{2}\left(|01\rangle ⊗|E_{1}\rangle \right)=e_{0}|00\rangle |k_{A0}\rangle +e_{1}|01\rangle |k_{A1}\rangle +e_{2}|10\rangle |k_{A2}\rangle +e_{3}|11\rangle |k_{A3}\rangle \;U_{2}\left(|10\rangle ⊗|E_{1}\rangle \right)=f_{0}|00\rangle |l_{A0}\rangle +f_{1}|01\rangle |l_{A1}\rangle +f_{2}|10\rangle |l_{A2}\rangle +f_{3}|11\rangle |l_{A3}\rangle$$

The initial state of Eve’s probe qubit denotes $|E_{i}\rangle$. $|g_{A0}\rangle ,|g_{A1}\rangle ,|g_{A2}\rangle ,|g_{A3}\rangle ,|h_{A0}\rangle ,|h_{A1}\rangle ,|h_{A2}\rangle ,|h_{A3}\rangle ,|i_{A0}\rangle ,|i_{A1}\rangle ,|i_{A2}\rangle ,|i_{A3}\rangle ,|j_{A0}\rangle ,|j_{A1}\rangle ,|j_{A2}\rangle ,|j_{A3}\rangle ,$$|k_{A0}\rangle ,|k_{A1}\rangle ,|k_{A2}\rangle ,|k_{A3}\rangle ,|l_{A0}\rangle ,|l_{A1}\rangle ,|l_{A2}\rangle \;\mathrm{and}\;|l_{A3}\rangle$ are distinguished by Bob, where

$$\begin{array}{ll} {\left|a_{0}\right|}^{2}+{\left|a_{1}\right|}^{2}+{\left|a_{2}\right|}^{2}+{\left|a_{3}\right|}^{2} & ={\left|b_{0}\right|}^{2}+{\left|b_{1}\right|}^{2}+{\left|b_{2}\right|}^{2}+{\left|b_{3}\right|}^{2}={\left|c_{0}\right|}^{2}+{\left|c_{1}\right|}^{2}+{\left|c_{2}\right|}^{2}+{\left|c_{3}\right|}^{2}\\ & ={\left|d_{0}\right|}^{2}+{\left|d_{1}\right|}^{2}+{\left|d_{2}\right|}^{2}+{\left|d_{3}\right|}^{2}={\left|e_{0}\right|}^{2}+{\left|e_{1}\right|}^{2}+{\left|e_{2}\right|}^{2}+{\left|e_{3}\right|}^{2}\\ & ={\left|f_{0}\right|}^{2}+{\left|f_{1}\right|}^{2}+{\left|f_{2}\right|}^{2}+{\left|f_{3}\right|}^{2}=1 \end{array}$$

To demonstrate the attack clearly, assume Alice chooses $|k^{+}\rangle =\frac{1}{2}\left(|010\rangle +|001\rangle +\sqrt{2}|100\rangle \right)$ as the initial state. It should be noted that the choice of initial state does not affect the security analysis. Suppose Eve performs the *U*_1_ operation, the possibilities are described as follows:

$$U_{1}\left({|k^{+}\rangle }_{ABC}⊗|E_{1}\rangle \right)=\frac{1}{2}\left(\begin{array}{c} {|0\rangle }_{A}⊗\left(c_{0}{|00\rangle }_{BC}|i_{A0}\rangle +c_{1}{|01\rangle }_{BC}|i_{A1}\rangle +c_{2}{|10\rangle }_{BC}|i_{A2}\rangle +c_{3}{|11\rangle }_{BC}|i_{A3}\rangle \right)+\\ {|0\rangle }_{A}⊗\left(b_{0}|00\rangle |h_{A0}\rangle +b_{1}|01\rangle |h_{A1}\rangle +b_{2}|10\rangle |h_{A2}\rangle +b_{3}|11\rangle |h_{A3}\rangle \right)+\\ \sqrt{2}({|1\rangle }_{A}⊗\left(a_{0}{|00\rangle }_{BC}|g_{A0}\rangle +a_{1}{|01\rangle }_{BC}|g_{A1}\rangle +a_{2}{|10\rangle }_{BC}|g_{A2}\rangle +a_{3}{|11\rangle }_{BC}|g_{A3}\rangle )\right) \end{array}\right)\;=\frac{1}{2}((c_{0}{|000\rangle }_{ABC}|i_{A0}\rangle +c_{1}{|001\rangle }_{ABC}|i_{A1}\rangle +c_{2}{|010\rangle }_{ABC}|i_{A2}\rangle +c_{3}{|011\rangle }_{ABC}|i_{A3}\rangle )\;+\left(b_{0}{|000\rangle }_{ABC}|h_{A0}\rangle +b_{1}{|001\rangle }_{ABC}|g_{A1}\rangle +b_{2}{|010\rangle }_{ABC}|g_{A2}\rangle +b_{3}{|011\rangle }_{ABC}|g_{A3}\rangle \right)\;+\sqrt{2}\left(a_{0}{|100\rangle }_{ABC}|g_{A0}\rangle +a_{1}{|101\rangle }_{ABC}|g_{A1}\rangle +a_{2}{|110\rangle }_{ABC}|g_{A2}\rangle +a_{3}{|111\rangle }_{ABC}|g_{A3}\rangle ))\right)\;\frac{1}{2}\left(\begin{array}{c} {|000\rangle }_{ABC}⊗\left(c_{0}|i_{A0}\rangle +b_{0}|h_{A0}\rangle )\right)+\\ {|001\rangle }_{ABC}⊗\left(c_{1}|i_{A1}\rangle +b_{1}|g_{A1}\rangle \right)+\\ {|010\rangle }_{ABC}⊗\left(c_{2}|i_{A2}\rangle +b_{2}|g_{A2}\rangle \right)+\\ {|011\rangle }_{ABC}⊗\left(c_{3}|i_{A3}\rangle +b_{3}|g_{A3}\rangle \right)+\\ \sqrt{2}({|100\rangle }_{ABC}⊗\left(a_{0}|g_{A0}\rangle \right)+\\ {|101\rangle }_{ABC}⊗\left(a_{1}|g_{A1}\rangle \right)+\\ {|110\rangle }_{ABC}⊗\left(a_{2}|g_{A2}\rangle \right)+\\ {|111\rangle }_{ABC}⊗\left(a_{3}|g_{A3}\rangle \right)) \end{array}\right)$$

Bob performs Z-basis measurement on the received qubits and saves the measurement results $MR_{CB}$ based on $K_{2}$. If the qubits are modified, $MR_{CB}$ will be altered and detected by Bob. Hence, the attack is assumed not to modify the value of the Z-basis qubits to pass the eavesdropping check. The restriction limits the possibilities of $U_{1}$ as follows:

$$U_{1}\left({|k^{+}\rangle }_{ABC}⊗|E_{1}\rangle \right)=\frac{1}{2}\left(\begin{array}{c} {|001\rangle }_{ABC}⊗\left(c_{1}|i_{A1}\rangle +b_{1}|g_{A1}\rangle \right)+\\ {|010\rangle }_{ABC}⊗\left(c_{2}|i_{A2}\rangle +b_{2}|g_{A2}\rangle \right)+\\ \sqrt{2}({|100\rangle }_{ABC}⊗\left(a_{0}|g_{A0}\rangle \right) \end{array}\right)$$

Then, Eve performs the second unitary operation $U_{2}$ on the qubits that are resent by Bob. The possibilities are described as follows:

$$U_{2}U_{1}({|k^{+}\rangle }_{ABC}⊗|E_{1}\rangle )=\frac{1}{2}\left(\begin{array}{c} {|0\rangle }_{A}⊗\left(e_{0}|00\rangle |k_{A0}\rangle +e_{1}|01\rangle |k_{A1}\rangle +e_{2}|10\rangle |k_{A2}\rangle +e_{3}|11\rangle |k_{A3}\rangle \right)⊗\left(c_{1}|i_{A1}\rangle +b_{1}|g_{A1}\rangle \right)+\\ {|0\rangle }_{A}⊗\left(f_{0}|00\rangle |l_{A0}\rangle +f_{1}|01\rangle |l_{A1}\rangle +f_{2}|10\rangle |l_{A2}\rangle +f_{3}|11\rangle |l_{A3}\rangle \right)⊗\left(c_{2}|i_{A2}\rangle +b_{2}|g_{A2}\rangle \right)+\\ \sqrt{2}\left({|1\rangle }_{A}⊗d_{0}|00\rangle |j_{A0}\rangle +d_{1}|01\rangle |j_{A1}\rangle +d_{2}|10\rangle |j_{A2}\rangle +d_{3}|11\rangle |j_{A3}\rangle ⊗\left(a_{0}|g_{A0}\rangle \right)\right) \end{array}\right)\;=\frac{1}{2}\left(\begin{array}{c} \left(e_{0}{|000\rangle }_{ABC}|k_{A0}\rangle +e_{1}{|001\rangle }_{ABC}|k_{A1}\rangle +e_{2}{|010\rangle }_{ABC}|k_{A2}\rangle +e_{3}{|011\rangle }_{ABC}|k_{A3}\rangle \right)⊗\left(c_{1}|i_{A1}\rangle +b_{1}|g_{A1}\rangle \right)+\\ \left(f_{0}{|000\rangle }_{ABC}|l_{A0}\rangle +f_{1}{|001\rangle }_{ABC}|l_{A1}\rangle +f_{2}{|010\rangle }_{ABC}|l_{A2}\rangle +f_{3}{|011\rangle }_{ABC}|l_{A3}\rangle \right)⊗\left(c_{2}|i_{A2}\rangle +b_{2}|g_{A2}\rangle \right)+\\ \sqrt{2}\left(d_{0}{|100\rangle }_{ABC}|j_{A0}\rangle +d_{1}{|101\rangle }_{ABC}|j_{A1}\rangle +d_{2}{|110\rangle }_{ABC}|j_{A2}\rangle +d_{3}{|111\rangle }_{ABC}|j_{A3}\rangle ⊗\left(a_{0}|g_{A0}\rangle \right)\right) \end{array}\right)\;=\frac{1}{2}\left(\begin{array}{c} \left(e_{0}{|000\rangle }_{ABC}|k_{A0}\rangle c_{1}|i_{A1}\rangle +e_{1}{|001\rangle }_{ABC}|k_{A1}\rangle c_{1}|i_{A1}\rangle +e_{2}{|010\rangle }_{ABC}|k_{A2}\rangle c_{1}|i_{A1}\rangle +e_{3}{|011\rangle }_{ABC}|k_{A3}\rangle c_{1}|i_{A1}\rangle \right)+\\ \left(e_{0}{|000\rangle }_{ABC}|k_{A0}\rangle b_{1}|g_{A1}\rangle +e_{1}{|001\rangle }_{ABC}|k_{A1}\rangle b_{1}|g_{A1}\rangle +e_{2}{|010\rangle }_{ABC}|k_{A2}\rangle b_{1}|g_{A1}\rangle +e_{3}{|011\rangle }_{ABC}|k_{A3}\rangle b_{1}|g_{A1}\rangle \right)\\ \left(f_{0}{|000\rangle }_{ABC}|l_{A0}\rangle c_{2}|i_{A2}\rangle +f_{1}{|001\rangle }_{ABC}|l_{A1}\rangle c_{2}|i_{A2}\rangle +f_{2}{|010\rangle }_{ABC}|l_{A2}\rangle c_{2}|i_{A2}\rangle +f_{3}{|011\rangle }_{ABC}|l_{A3}\rangle c_{2}|i_{A2}\rangle \right)+\\ \left(f_{0}{|000\rangle }_{ABC}|l_{A0}\rangle b_{2}|g_{A2}\rangle +f_{1}{|001\rangle }_{ABC}|l_{A1}\rangle b_{2}|g_{A2}\rangle +f_{2}{|010\rangle }_{ABC}|l_{A2}\rangle b_{2}|g_{A2}\rangle +f_{3}{|011\rangle }_{ABC}|l_{A3}\rangle b_{2}|g_{A2}\rangle \right)+\\ \sqrt{2}\left(d_{0}{|100\rangle }_{ABC}|j_{A0}\rangle a_{0}|g_{A0}\rangle +d_{1}{|101\rangle }_{ABC}|j_{A1}\rangle a_{0}|g_{A0}\rangle +d_{2}{|110\rangle }_{ABC}|j_{A2}\rangle a_{0}|g_{A0}\rangle +d_{3}{|111\rangle }_{ABC}|j_{A3}\rangle a_{0}|g_{A0}\rangle \right) \end{array}\right)\;=\frac{1}{2}\left(\begin{array}{c} {|000\rangle }_{ABC}⊗\left(e_{0}|k_{A0}\rangle c_{1}|i_{A1}\rangle +e_{0}|k_{A0}\rangle b_{1}|g_{A1}\rangle +f_{0}|l_{A0}\rangle c_{2}|i_{A2}\rangle +f_{0}|l_{A0}\rangle b_{2}|g_{A2}\rangle \right)+\\ {|001\rangle }_{ABC}⊗\left(e_{1}|k_{A1}\rangle c_{1}|i_{A1}\rangle +e_{1}|k_{A1}\rangle b_{1}|g_{A1}\rangle +f_{1}|l_{A1}\rangle c_{2}|i_{A2}\rangle +f_{1}|l_{A1}\rangle b_{2}|g_{A2}\rangle \right)+\\ {|010\rangle }_{ABC}⊗\left(e_{2}|k_{A2}\rangle c_{1}|i_{A1}\rangle +e_{2}|k_{A2}\rangle b_{1}|g_{A1}\rangle +f_{2}|l_{A2}\rangle c_{2}|i_{A2}\rangle +f_{2}|l_{A2}\rangle b_{2}|g_{A2}\rangle \right)+\\ {|011\rangle }_{ABC}⊗\left(e_{3}|k_{A3}\rangle c_{1}|i_{A1}\rangle +e_{3}|k_{A3}\rangle b_{1}|g_{A1}\rangle +f_{3}|l_{A3}\rangle c_{2}|i_{A2}\rangle +f_{3}|l_{A3}\rangle b_{2}|g_{A2}\rangle \right)+\\ \sqrt{2}({|100\rangle }_{ABC}⊗\left(d_{0}|j_{A0}\rangle a_{0}|g_{A0}\rangle \right)+\\ {|101\rangle }_{ABC}⊗\left(d_{1}|j_{A1}\rangle a_{0}|g_{A0}\rangle \right)+\\ {|110\rangle }_{ABC}⊗\left(d_{2}|j_{A2}\rangle a_{0}|g_{A0}\rangle \right)+\\ {|111\rangle }_{ABC}⊗(d_{3}|j_{A3}\rangle a_{0}|g_{A0}\rangle ) \end{array}\right)$$

Alice receives the photons which are resent by Bob and performs the eavesdropping check. If the quantum state is not equal to W states, Alice will detect the attack. To pass the eavesdropping check, suppose $e_{0}|k_{A0}\rangle c_{1}|i_{A1}\rangle +e_{0}|k_{A0}\rangle b_{1}|g_{A1}\rangle +f_{0}|l_{A0}\rangle c_{2}|i_{A2}\rangle +f_{0}|l_{A0}\rangle b_{2}|g_{A2}\rangle =e_{3}|k_{A3}\rangle c_{1}|i_{A1}\rangle +e_{3}|k_{A3}\rangle b_{1}|g_{A1}\rangle +f_{3}|l_{A3}\rangle c_{2}|i_{A2}\rangle +f_{3}|l_{A3}\rangle b_{2}|g_{A2}\rangle =d_{1}|j_{A1}\rangle a_{0}|g_{A0}\rangle =d_{2}|j_{A2}\rangle a_{0}|g_{A0}\rangle =d_{3}|j_{A3}\rangle a_{0}|g_{A0}\rangle =\vec{0}$. Hence, it is assumed that the attack cannot modify the value of the Z-basis photons. With the restriction mentioned above, limits $U_{2}$ possibilities as follows:

$$U_{2}U_{1}\left({|k^{+}\rangle }_{ABC}⊗|E_{1}\rangle \right)=\frac{1}{2}\left(\begin{array}{c} {|001\rangle }_{ABC}⊗\left(e_{0}|k_{A0}\rangle c_{1}|i_{A1}\rangle +e_{0}|k_{A0}\rangle b_{1}|g_{A1}\rangle +f_{0}|l_{A0}\rangle c_{2}|i_{A2}\rangle +f_{0}|l_{A0}\rangle b_{2}|g_{A2}\rangle \right)+\\ {|010\rangle }_{ABC}⊗\left(e_{2}|k_{A2}\rangle c_{1}|i_{A1}\rangle +e_{2}|k_{A2}\rangle b_{1}|g_{A1}\rangle +f_{2}|l_{A2}\rangle c_{2}|i_{A2}\rangle +f_{2}|l_{A2}\rangle b_{2}|g_{A2}\rangle \right)+\\ \sqrt{2}({|100\rangle }_{ABC}⊗\left(d_{0}|j_{A0}\rangle a_{0}|g_{A0}\rangle \right) \end{array}\right)$$

Suppose Eve wants to pass the eavesdropping check, Eve must set all the measurement result of probe qubits $|E_{1}\rangle$ to be equal (i.e., $e_{0}|k_{A0}\rangle c_{1}|i_{A1}\rangle +e_{0}|k_{A0}\rangle b_{1}|g_{A1}\rangle +f_{0}|l_{A0}\rangle c_{2}|i_{A2}\rangle +f_{0}|l_{A0}\rangle b_{2}|g_{A2}\rangle =e_{2}|k_{A2}\rangle c_{1}|i_{A1}\rangle +e_{2}|k_{A2}\rangle b_{1}|g_{A1}\rangle +f_{2}|l_{A2}\rangle c_{2}|i_{A2}\rangle +f_{2}|l_{A2}\rangle b_{2}|g_{A2}\rangle =d_{0}|j_{A0}\rangle a_{0}|g_{A0}\rangle$). Without altering ${|k^{+}\rangle }_{ABC}$, Eve can pass the eavesdropping check. In contrast, Eve cannot distinguish the corresponding measurement result of $|E_{1}\rangle$. Hence, Eve cannot deduce any useful information. On the other hand, suppose Eve wants to deduce the information from the measurement result of probe qubits $|E_{1}\rangle$, Eve must set all the measurement results of probe qubits $|E_{1}\rangle$ not to be equal, so Eve can distinguish the corresponding result. In contrast, Eve will get detected by the eavesdropping check because the value of ${|k^{+}\rangle }_{ABC}$ is altered. Thus, the proposed ASQKD protocol is proven to be immune to the collective attack. □

### 5.5. Key Leakage Problem

Assume Eve tries to eavesdrop on the raw key from the traveling qubits. Eve may perform Z-basis measurement on the photon sequence sent by Alice, $W_{2}$ and $W_{3}$. Eve obtains the measurement results of $W_{2}$ and $W_{3}$ (i.e., |00〉,|01〉,|10〉,|11〉), which implies the raw key (i.e., “00”, “01”, “10”, “11”). Suppose Shannon entropy is defined as $E=-\Sigma_{i}\rho i\log_{2}\rho i$, where ρi denotes probability distribution. The entropy $E_{1}$ can be computed as $E_{1}=-4\times \frac{1}{4}\log_{2}\frac{1}{4}=2$ bits. However, the protocol provides an eavesdropping check, which limits the possibility of the measurement results of $W_{2}$ and $W_{3}$ being used as the raw key, hence the probability is $\frac{1}{4}$ (i.e., Bob receives $W_{2}$ and $W_{3}$ and performs measure-resend or reflect based on $K_{2}$. If $K_{2}=0$, Bob records the measurement results as $MR_{CB}$ or $MR_{KB}$. If $K_{2}=1\;\mathrm{or}$ 2, Bob measure-resend or reflect for eavesdropping check. Assume Alice and Bob consume half of the transmitted photons as eavesdropping check, hence $K_{2}=0$ and $K_{2}=1\;\mathrm{or}$ 2 consume each half of the measurement results. While in $K_{2}=0$, the measurement results of $K_{3}=0$ denote as check bit, $K_{3}=1$ denote as key bit, thus half of the $K_{2}=0$ measurement results are used as sharing raw key. Eventually, the probability of Eve eavesdrops the raw key from the measurement results of $W_{2}$ and $W_{3}$ is $\frac{1}{2}\times \frac{1}{2}=\frac{1}{4}$). Hence, the entire entropy denotes $\frac{1}{4}\times E_{1}=0.5$ bit. Even though Eve can obtain 0.5 bit by performing eavesdropping, eventually the attack will be detected by an eavesdropping check. Even if Eve passes the eavesdropping check, one can still perform the privacy amplification process [67,68] on the transmitted information to distill the private key, avoiding the key leakage problem. Thus, Eve cannot obtain any private key under an eavesdropping attack.

## 6. Efficiency Analysis

Table 3 provides a comparison of the Yu et al. [59], Li et al. [60], Zebboudj et al. [63], Chang et al. [64], and Wang et al. [65], and Wen et al.’s [66] measure-resend ASQKD protocols with the proposed ASQKD protocol. The efficiency of the protocol is calculated using the following equation: $\eta =\frac{c}{q}$, where c denotes the number of shared classical bits and q denotes the sum of consumed qubits. We assume that Alice generates a binary string of length *n* as the secret key. The length of the hash, decoy photon, and checking bit *m* is assumed to equal that of the secret key (i.e., *n* = *m*).

The efficiency analysis of Yu et al., Li et al., Zebboudj et al., Chang et al., and Wang et al.’s ASQKD protocol have been discussed in Wang et al.’s study [65]. For clarity and readability, this study is briefly summarized as follows: the efficiency of Yu et al., Li et al., Zebboudj et al., Chang et al., and Wang et al.’s ASQKD protocol is 10%, 11%, 14%, 17%, 14%, respectively.

In Wen et al.’s ASQKD protocol, Alice generated *n* GHZ-like states (i.e., 3*n* qubits) and *n* decoy states. She then generates 3*n* W states (i.e., 9*n* qubits) for teleportation of the state |Y〉 and decoy state $|\varphi_{L}\rangle$. Bob measures and generates $|Y^{\prime }\rangle$ (i.e., *n* qubits). Thus, the efficiency of the ASQKD protocol proposed by Wen et al. is $\eta =\frac{n}{3n+n+9n+n}=\frac{1}{14}\approx 7\%$.

In the proposed ASQKD protocol, Alice prepares four pairs of W states (i.e., 12*n* qubits), Bob measures the second and third W states and generates three pairs of two single photons (i.e., based on $K_{0}$ generates *2n + 2n* qubits, $K_{1}$ generates *2n* qubits). Eventually, Alice and Bob share a secret key of 2*n* bits. Hence, the efficiency of the proposed ASQKD protocol is $\eta =\frac{2n}{12n+2n+2n+2n}=\frac{1}{9}\approx 11\%$.

The proposed ASQKD protocol improves the malfunction issue in the ASQKD protocol of Wen et al. [66]. As mentioned in Section 3.1, in Wen et al.’s ASQKD protocol, Bob cannot perform the teleportation due to the insufficient information on the corresponding position of |Y〉 and $|\varphi_{L}\rangle$. The proposed ASQKD protocol pre-shares keys for the information of the photon’s position privately, ensuring that the unfunctional situation with Bob will not occur under all circumstances.

As mentioned in Section 3.2, in Wen et al.’s ASQKD protocol [66], Bob must preserve all photons sent by Alice in Step W1 for the measurements and calculations to perform the teleportation of W states later. This implies that Bob must possess quantum memory, which strongly disobeys the definition of semi-quantum environment [17]. In the proposed ASQKD protocol, Alice sends the photons individually to Bob, and Bob performs measurements or reflects the photons as he receives them. The proposed ASQKD protocol ensures that Bob does not need to equip quantum memory, which is a legal semi-quantum environment.

From the perspective of entangle states applying ASQKD protocols, the proposed ASQKD protocol provided higher qubit efficiency compared to Yu et al. [59] and Li et al. [60]. Although Chang et al. [64] have higher qubit efficiency, their protocol suffers from the reflecting attack [65], the protocol must apply more qubits to secure the channel. Thus, Chang et al. [64] provided lower qubit efficiency than the proposed ASQKD protocol. Zebboudj et al. [63] and Wang [65] take advantages in qubit efficiency, however, the security of those protocols is based on classical cryptography, the mathematics of the hash function. With the advance of quantum computing, powerful quantum computing may be a potential menace to classical cryptography. The proposed protocol does not require the hash function, thus is secured even in the future.

From the perspective of quantum hardware, in Wen et al.’s ASQKD protocol [66], Alice generates GHZ-like states and W states, and Bob equips quantum memory, which requires advanced quantum mechanics. In the proposed ASQKD protocol, Alice generates only W states, and Bob does not require quantum memory, which is more practical. Moreover, the proposed ASQKD protocol has higher efficiency than Wen et al.’s ASQKD protocol.

By combining all the benefits mentioned above, the proposed ASQKD protocol reduces the quantum hardware requirement and elevates the efficiency significantly when equipped with a secure legal semi-quantum environment. When compared to Wen et al.’s ASQKD protocol [66], the proposed ASQKD protocol has several advantages.

1. The proposed ASQKD protocol ensures the procedure is functional.
2. The proposed ASQKD protocol does not require quantum memory and legally fulfills a semi-quantum environment [17].
3. The proposed ASQKD protocol, based on W states, only reduces the quantum hardware requirements.
4. The qubit efficiency of the proposed ASQKD protocol is 1.6 times higher than that of Wen et al.’s ASQKD protocol.
5. The proposed ASQKD protocol does not require classical cryptography (i.e., the hash function), which does not show the potential menace of the advance quantum computing.

## 7. Conclusions

In this study, several important issues in Wen et al.’s ASQKD protocol were addressed and an improvement was proposed. In 2019, Wen et al. proposed an ASQKD protocol for identity and messages using the teleportation of W states and GHZ-like states without pre-shared keys. However, Wen et al.’s ASQKD protocol exhibits significant design flaws. The teleportation of W states in Wen et al.’s ASQKD protocol was malfunctioning. Moreover, Wen et al.’s ASQKD protocol requires the classical user to equip quantum memory, which strongly disobeys the definition of the semi-quantum environment defined by Boyer et al. Therefore, in this study, we proposed an ASQKD protocol based only on the W states. When compared with Wen et al.’s ASQKD protocol, the proposed ASQKD protocol circumvented the aforementioned flaws and obtained the following advantages: runnable ASQKD protocol, legal semi-quantum environment (i.e., does not require quantum memory), reduced quantum hardware requirement (i.e., based only on W states), does not involve classical cryptography (i.e., the hash function) and provided 1.6 times higher qubit efficiency, which significantly elevated security and efficiency. To obtain a higher efficiency in the ASQKD protocol, further research is required.

## Figures and Tables

**Table 1:** Coding rule of generating L.

|Y〉=|0〉	|Y〉=|1〉	|Y〉=|+〉	|Y〉=|−〉
L=00	L=01	L=10	L=11

**Table 2:** Coding rule of generating $M_{3}^{\prime }$.

	$m_{3}=0$	$m_{3}=1$
$\kappa^{+}$	$m_{3}^{\prime }=0$	$m_{3}^{\prime }=1$
$\kappa^{-}$	$m_{3}^{\prime }=0$	$m_{3}^{\prime }=1$
$\gamma^{+}$	$m_{3}^{\prime }=1$	$m_{3}^{\prime }=0$
$\gamma^{-}$	$m_{3}^{\prime }=1$	$m_{3}^{\prime }=0$

**Table 3:** Comparison of [59,60,63,64,65,66] and the proposed ASQKD protocol.

	Yu et al.’s ASQKD Protocol [59]	Li et al.’s ASQKD Protocol [60]	Zebboudj et al.’s ASQKD Protocol [63]	Chang et al.’s ASQKD Protocol [64]	Wang et al.’s ASQKD Protocol [65]	Wen et al.’s ASQKD Protocol [66]	The Proposed ASQKD Protocol
Quantum resource	Bell states	Bell states, Single photons	Single photons	Single photons	Single photons	GHZ-like states W states	W states
Qubit efficiency	10%	11%	14%	17%	14%	7%	11%
Required pre-shared keys (in bits)	6n	4n	3n	3n	3n	4n	5n
Classical participant’s quantum capabilities	Generate Reflect Measure	Generate Reflect Measure	Generate Reflect Measure	Generate Reflect Measure	Generate Reflect Measure	Generate Reflect Measure Quantum memory	Generate Reflect Measure
Classical participant does not require quantum memory	Yes	Yes	Yes	Yes	Yes	No	Yes
Legal semi-quantum environment	Yes	Yes	Yes	Yes	Yes	No	Yes
The protocol does not require the hash function	Yes	No	No	No	No	No	Yes
Runnable protocol	Yes	Yes	Yes	Yes	Yes	No	Yes
Required classical channel	Yes	Yes	Yes	Yes	No	Yes	Yes
Robustness of the reflecting attack	Yes	Yes	Yes	No	Yes	Yes	Yes

## Data Availability

Not applicable.
